# Long-Term Outcome of Otherwise Healthy Individuals with Incidentally Discovered Borderline Thrombocytopenia

**DOI:** 10.1371/journal.pmed.0030024

**Published:** 2006-01-17

**Authors:** Roberto Stasi, Sergio Amadori, John Osborn, Adrian C Newland, Drew Provan

**Affiliations:** **1**Department of Medical Sciences, Ospedale “Regina Apostolorum,” Albano Laziale, Rome, Italy; **2**Department of Hematology, University of Rome “Tor Vergata,” Rome, Italy; **3**Department of Public Health Science, University of Rome “La Sapienza,” Rome, Italy; **4**Department of Haematology, St Bartholomew's & The Royal London School of Medicine & Dentistry, London, United Kingdom; University of South FloridaUnited States of America

## Abstract

**Background:**

The long-term outcome of individuals with mild degrees of thrombocytopenia is unknown.

**Methods and Findings:**

In a prospective study conducted between August 1992 and December 2002, 260 apparently healthy individuals with incidentally discovered platelet counts between 100 × 10^9^/l and 150 × 10^9^/l were monitored for 6 mo to determine whether their condition persisted. The monitoring period was completed in 217 cases, of whom 191 (88%) maintained stable platelet counts. These 191 individuals were included in a long-term follow-up study to gain knowledge of their natural history. With a median time of observation of 64 mo, the thrombocytopenia resolved spontaneously or persisted with no other disorders becoming apparent in 64% of cases. The most frequent event during the study period was the subsequent development of an autoimmune disease. The 10-y probability of developing idiopathic thrombocytopenic purpura (ITP), as defined by platelet counts persistently below 100 × 10^9^/l, was 6.9% (95% confidence interval [CI]: 4.0%–12.0%). The 10-y probability of developing autoimmune disorders other than ITP was 12.0% (95% CI: 6.9%–20.8%). Most of the cases (85%) of autoimmune disease occurred in women.

**Conclusions:**

Healthy individuals with a sustained platelet count between 100 × 10^9^/l and 150 × 10^9^/l have a 10-y probability of developing autoimmune disorders of 12%. Further investigation is required to establish whether this risk is higher than in the general population and whether an intensive follow-up results in an improvement of prognosis.

## Abstract 2

A prospective study of 260 apparently healthy individuals with incidentally discovered platelet counts between 100 × 10^9^/l and 150 × 10^9^/l showed that there was a 10-year probability of 12% of developing autoimmune disorders.

## Introduction

With extensive automation in laboratories, an ever increasing number of asymptomatic individuals with platelet counts ranging between 100 × 10^9^/l and 150 × 10^9^/l are now being recognized [1–3]. However, both the clinical features of these individuals, as well as the natural history of their thrombocytopenia, have not been systematically studied. An undetermined number may go on to develop an overt disease associated with a low platelet count, others may maintain normal or borderline platelet counts indefinitely, but to date no consistent figures concerning these events are available.

Although the differential diagnosis is fairly broad, most asymptomatic adults who have a low platelet count as the sole laboratory abnormality are likely to have an immune thrombocytopenia, either primary (idiopathic) or secondary to an autoimmune disorder [4]. In fact, thrombocytopenia may often be the initial manifestation of a systemic autoimmune disease; thus, it may not be possible to differentiate secondary immune thrombocytopenia at the time of initial presentation from idiopathic thrombocytopenic purpura (ITP). Information about these patients has a potential clinical impact because many systemic autoimmune diseases can be treated and controlled if detected in the early stages. Patients often have symptoms for several years before the correct diagnosis is made, and this delay in treatment may cause damage to major organs and result in permanent disability [5].

This study was designed with the aim of elucidating the natural history of apparently healthy adults who were diagnosed with a platelet count between 100 × 10^9^/l and 150 × 10^9^/l, cases that in this study were defined as having “borderline thrombocytopenia.”

## Methods

### Patient Selection

This study was conducted prospectively between August 1992 and December 2002 and included a consecutive series of apparently healthy individuals who were referred to the outpatient clinics of the Department of Hematology of the University of Rome “Tor Vergata” and the Department of Medical Sciences of Ospedale “Regina Apostolorum” in Albano Laziale, Rome, because they had been found with a platelet count between 100 × 10^9^/l and 150 × 10^9^/l. Together, these two tertiary health-care centers serve a population of approximately 600,000 people in the southeast region of Rome and its surroundings. To be considered “apparently healthy,” such individuals had to be free of a history of chronic medical disorders such as hypertension, autoimmune disorders, liver diseases, and malignancies previously treated with chemotherapy or radiotherapy, and were not currently on medication or had not taken any medication in the last 3 mo; pregnancy had to be excluded in premenopausal women. Medical conditions that did not preclude inclusion in the study were iron-deficiency anemia (in menstruating women), thalassemia trait, and osteoarthritis.

In addition to obtaining a detailed clinical history, the initial evaluation of these patients included a physical examination and a complete blood count. The platelet count was determined using electronic analyzers, and was always confirmed by direct observation of peripheral blood smears. Additional laboratory tests included routine serum chemistry (renal and liver function, bone biochemistry), serum protein electrophoresis, antinuclear antibodies (ANA), antithyroperoxidase antibodies (TPO-Ab), anticardiolipin antibodies (ACA), screening for hepatitis B and C infection, screening for human immunodeficiency virus (HIV) infection, and rheumatoid factor. Ultrasound scans were performed in very obese people, in whom physical examination could not reliably exclude splenomegaly, as well as in those with positive ANA or rheumatoid factor.

### Study Design

Eligible patients were monitored for 6 mo with monthly visits, and if they maintained their platelet count between 100 × 10^9^/l and 150 × 10^9^/l, they were classified as “borderline thrombocytopenia.” Observation of these cases after the first 6 mo was scheduled every 3 mo for the first 2 y, and every 6 mo thereafter, unless there were clinical indications for more frequent visits. A complete blood count and clinical evaluation were performed at each visit.

It has been shown that autoantibodies are typically present many years before the diagnosis of an autoimmune disease is made [6,7]. To rule out the possibility that our study cohort was not a selected high-risk subset relative to the general population, we compared the autoantibody profile of these individuals with that of a control group. This control group comprised healthy individuals with platelet counts in excess of 150 × 10^9^/l, matched by sex and age (±2 y) to individuals of the study cohort (three controls for each individual of the study cohort). The control group was extracted from a larger sample of approximately 62,000 individuals in a population-based survey of metabolic disorders which had aimed to define the prevalence of diabetes, dyslipidemia, and hyperuricemia in the local population. An institutional review board–approved informed consent was obtained for both the study cohort and controls.

### Statistical Methods

Statistical analysis was carried out using the NCSS software package (NCSS, Kaysville, Utah, United States). The statistical significance of differences was evaluated by the chi-square test for categorical variables, whereas Student's *t-*test was used for continuous variables. A *p-*value of 0.05 or less was deemed to be statistically significant. The probability of developing either ITP or an autoimmune disease over time was estimated by the cumulative incidence procedure. Cumulative incidence, also referred to as competing risk analysis, is an extension of the Kaplan–Meier survival technique to the case where failure occurs for several reasons [8,9]. The Kaplan–Meier approach provides a nonparametric estimate of the overall survival probability of an event of interest, but ignores the presence of competing risks. In contrast, the cumulative incidence estimates the percentage of patients who will be diagnosed with the event of interest in a certain time interval in the presence of competing risks. In our case, the event of interest was the development of either ITP or an autoimmune disease, and competing causes were considered: lost to follow-up (including cases who had achieved a sustained normal platelet count), death, development of either an autoimmune disease (when the cumulative incidence of ITP was estimated) or of ITP (when the cumulative incidence of an autoimmune disease was estimated), and development of other diseases (cardiovascular diseases, malignancies, or myelodysplastic syndromes).

The effect of the presence of autoimmune markers on the risk of developing autoimmune disorders was estimated by proportional hazards regression analysis.

The analysis was based on follow-up information obtained through November 30, 2004.

## Results

### Study Cohort Characteristics

Five-hundred twenty-seven individuals with a platelet count between 100 × 10^9^/l and 150 × 10^9^/l and no other hematologic abnormalities apart from iron-deficiency anemia (in menstruating women) or thalassemia trait were seen in our outpatient clinics during the study period. Two hundred fifty-four were receiving continuous medication that could not be discontinued or had a history of malignancy or chronic inflammatory disorder, and were excluded from this study. Of the remaining 273, 13 were also excluded for the following reasons: Three tested positive for hepatitis C virus, one was a chronic carrier of hepatitis B surface antigen, five had hypothyroidism associated with high titers of TPO-Ab, and five were diagnosed with Sjögren's Syndrome.

Markers of autoimmunity in the absence of other clinical or laboratory abnormalities suggestive of an autoimmune disorder were found in 37 (14.2 %) cases. These individuals, as well as 223 others who had no laboratory abnormality, were considered eligible for the study. Thus, we began the 6-mo monitoring phase in 260 individuals (107 men and 153 women; median age 52 y, range 15–82 y). The study design and results are summarized in Figure 1.

**Figure 1:**
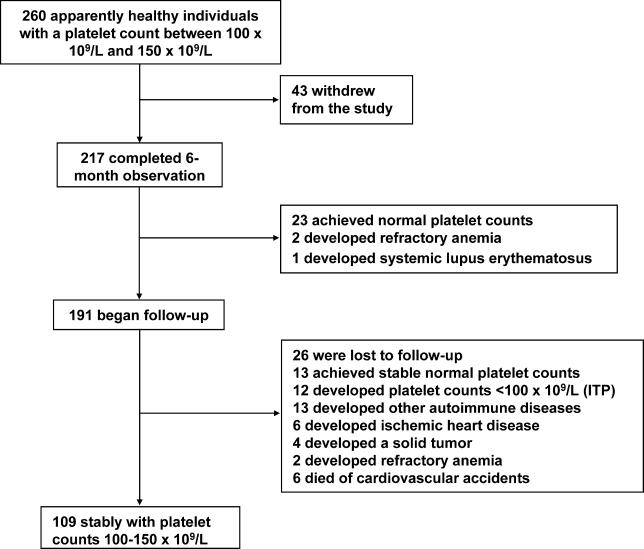
Study Design and Results

### Initial 6-mo Observation

The initial monitoring period of 6 mo was completed in 217 (83.4%) of 260 cases. The 43 participants who did not complete this phase communicated their withdrawal from the study with refusal to undergo medical assessments. Their clinical characteristics did not differ significantly from those of the patients who actually completed the initial follow-up (Table 1).

**Table 1:**
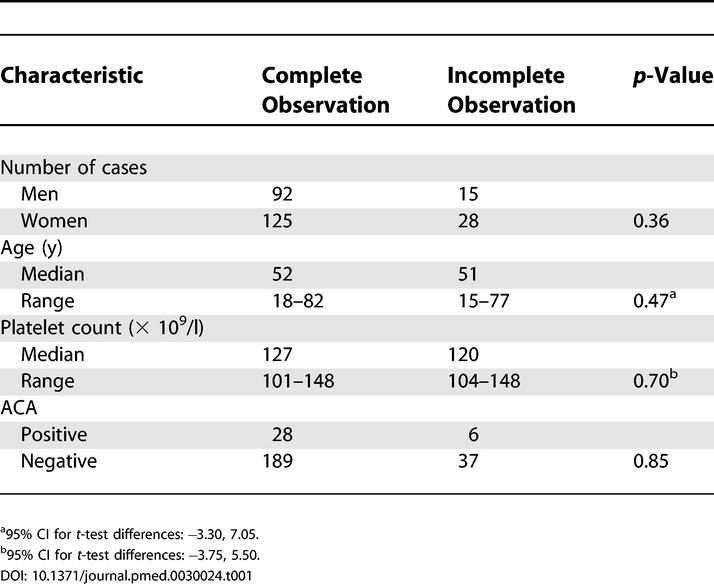
Comparisons of Clinical Characteristics between Individuals Who Completed the Observation Phase and Those Who Withdrew from Study before 6 mo

Among those who completed this initial observation period, a 73-y-old woman and a 69-y-old man had worsening thrombocytopenia with subsequent development of anemia. They were diagnosed with a myelodysplastic syndrome (refractory anemia) after 3 and 5 mo of observation, respectively. A 32-y-old woman who tested positive for both ANA and ACA developed hypertension and renal function abnormalities, and was diagnosed with systemic lupus erythematosus after 5 mo of observation. The other individuals had a variable pattern of their platelet counts (Table 2). There were no significant differences in terms of gender, age, autoimmune profile, or baseline platelet count between these various groups of individuals.

**Table 2:**
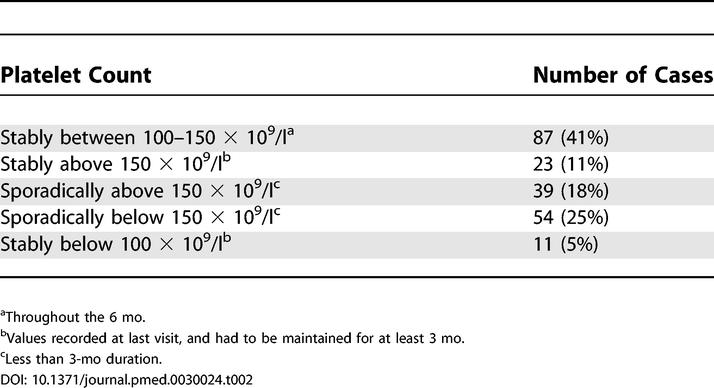
Behavior of the Platelet Counts during the Initial 6-mo Monitoring Phase in 214 Individuals Who Completed the Observation

A normal count that had been maintained for at least 3 mo was achieved by 23 individuals. Although these cases were followed for three more months to confirm the stability of the platelet count, they were considered ineligible for the follow up study. In 11 individuals, platelet counts fell below 100 × 10^9^/l for at least 3 mo. In one case, the platelet count reached values as low as 33 × 10^9^/l. Nadir values for the other cases of this subset were between 47 × 10^9^/l and 83 × 10^9^/l. Bone marrow examination was performed in all of these patients to exclude myelodysplasia or other hematologic disorders. These cases were classified by default as ITP, but continued the observational study.

### Follow-Up

A total of 191 individuals with a confirmed borderline thrombocytopenia entered the follow-up study. Their baseline clinical and laboratory characteristics are reported in Table 3.

**Table 3:**
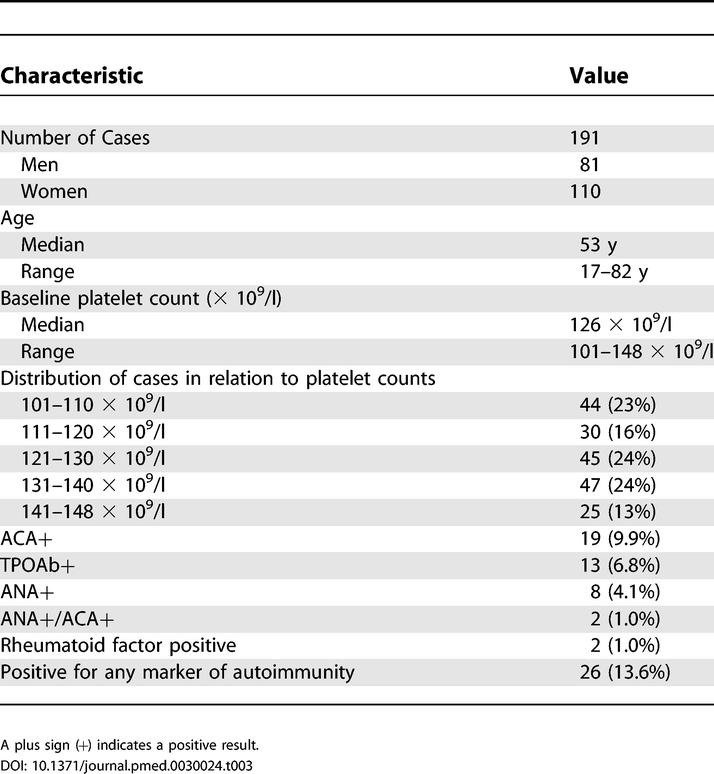
Characteristics of Individuals with Borderline Thrombocytopenia Who Began Follow-Up

In the control group, markers of autoimmunity were found in 70/573 (12.3%) individuals, a prevalence similar to that of the study cohort (13.6%; *p* = 0.614). Specifically, in controls, ACA were found in 46 cases (8.1%), high titers of TPO-Ab were detected in 36 (6.2%), ANA was positive in 16 (2.8%), and five had high levels of rheumatoid factor (0.9%).

Table 4 summarizes the outcome of individuals with borderline thrombocytopenia. The overall median follow-up duration was 64 mo (range 6–140 mo). Twenty-six individuals were lost to follow-up while their counts were stable above 100 × 10^9^/l. The median follow-up duration for these cases was 45.5 mo (range 14–124 mo). Median follow-up duration from the date of accrual for the remaining 165 cases with complete observation was 67 mo (range 6–140 mo).

**Table 4:**
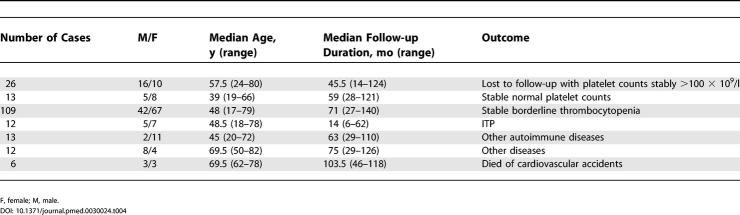
Outcome of Individuals with Borderline Thrombocytopenia Followed after the Initial 6-mo Observation Period

Thirteen individuals achieved stable (>6 mo) platelet counts above 150 × 10^9^/l. There was no association between the baseline platelet count and the probability of achieving a normal platelet count. One hundred nine individuals were considered to have a stable borderline thrombocytopenia. Although 93 of these individuals experienced fluctuations of their platelet counts below 100 × 10^9^/l, the duration of significant thrombocytopenia was never in excess of 4 mo.

Five of the 11 patients classified as having ITP after the initial 6-mo observation developed an autoimmune disease. Therefore, these cases were reclassified. Platelet counts below 100 × 10^9^/l developed in six other cases at 6 to 62 mo from the start of the study, with nadir values ranging from 14 to 65 × 10^9^/l. A bone marrow aspirate was performed in all these individuals, and was consistent with a diagnosis of having ITP. In total, at the end of the study, 12 cases met our definition of ITP, and the probability of developing ITP at 10 y was 6.9% (95% confidence interval [CI]: 4.0%–12.0%) (Figure 2).

**Figure 2:**
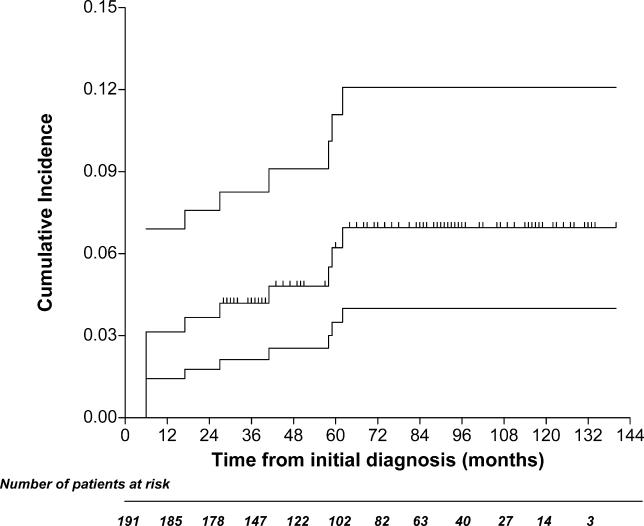
Cumulative Incidence of ITP The upper and lower lines indicate the 95% confidence limits.

An autoimmune disorder developed in 13 cases at 29 to 110 mo from the start of the study. Clinical symptoms were the cause that led 12 of these 13 participants to be further investigated for the underlying autoimmune disorder. In only one case (woman, age 72 y) was there the finding of abnormal liver function tests, in the absence of symptoms, which led to further investigation that resulted in the diagnosis of primary biliary cirrhosis. The detailed features for these individuals are reported in Table 5. It is noteworthy that in seven cases, markers of autoimmunity were present at the time they began follow-up.

**Table 5:**
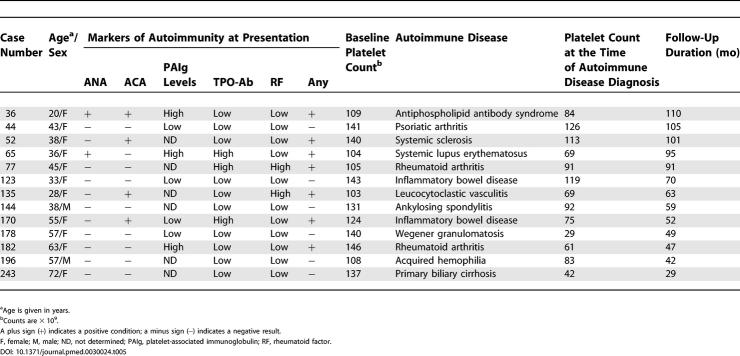
Clinical and Laboratory Characteristics of the 13 Individuals Who Developed an Autoimmune Disease during Follow-Up

The probability of developing autoimmune disorders other than ITP at 10 y was 12.0% (95% CI: 6.9%–20.8%) (Figure 3). The probability of developing autoimmune disorders among individuals who presented with autoimmune markers on study entry was 7.2% (95% CI: 4.0%–12.9%), and 4.7% (95% CI: 0.7%–12.2%) in those who had negative markers, with a hazard ratio of 2.78 (95% CI: 0.71–8.54; *p* = 0.117).

**Figure 3:**
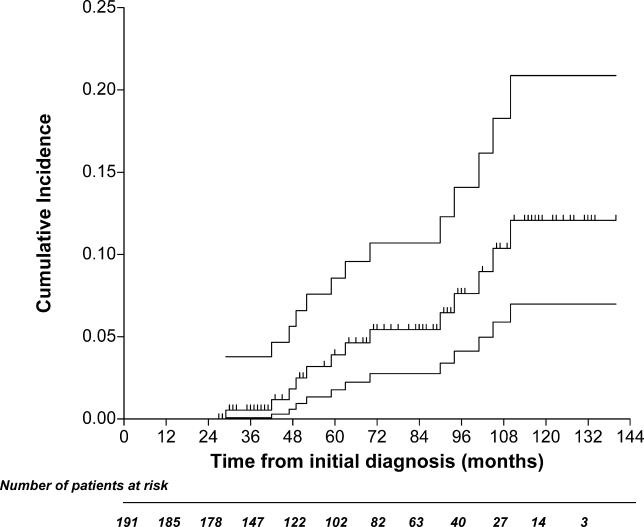
Cumulative Incidence of an Autoimmune Disease Other Than ITP The upper and lower lines indicate the 95% confidence limits.

Ischemic heart disease developed in six patients (four men and two women, median age 71.5 y) at 29 to 77 mo during follow-up. None of these patients had laboratory abnormalities consistent with an autoimmune disease.

A malignancy was diagnosed in six cases: myelodysplasia (refractory anemia) in one patient, breast cancer in two, colorectal cancer in one, and lung cancer in one. Finally, six additional individuals died. All deaths were attributed to cardiovascular accidents, but we were unable to obtain the details for these events.

## Discussion

In this study, 191 apparently healthy individuals with platelet counts stably between 100 × 10^9^/l and 150 × 10^9^/l for at least 6 mo were observed in order to determine their long-term outcome. The design of this study was stimulated by the paucity of data concerning the natural history of such individuals. It was particularly interesting to ascertain whether this condition represents a mild form of immune thrombocytopenia, either primary (idiopathic) or secondary to another autoimmune disorder that may manifest over time.

The methodology of the study deserves a few considerations. Although this was a prospective study involving a consecutive series of individuals, these cases probably represent only a small fraction of all cases in the areas served by our hospitals, which had incidentally discovered mild thrombocytopenia. In fact, many of these cases may not be referred to a hematologist. However, it would have been virtually impossible to include primary-care offices to capture all abnormal platelet counts in the region. The same issues of difficult practical approach apply to the follow-up of individuals in the control group. We are aware that follow-up of the control group would have been interesting to assess the relative importance of the follow-up observations in the study group. Unfortunately, attempts to involve healthy individuals to undergo long-term periodic medical assessment met with failure. Finally, the fact that individuals were only included if they were not currently on medication or had not taken any medication in the last 3 mo would appear to exclude the majority of elderly individuals. Nevertheless, we believe this is also one of the strengths of the study, which tried to eliminate all possible confounding factors. The dropout rate was 14%, which is perhaps not unexpected given the length of the study and the above cited reluctance of many healthy individuals to undergo medical assessment.

Interestingly, in the highly selected cohort of individuals that began the monitoring phase, a single borderline count was predictive of sustained borderline thrombocytopenia in 191 (88%) cases. Stably normal platelet counts were eventually achieved by 36 participants (14%), the majority of whom (23/36) improved their counts during the initial 6-mo observation period. This lends support to the hypothesis that these cases may have suffered from a “silent” viral infection or a minor insult of a different nature to the bone marrow. Another possibility is the seasonal variation of the platelet count that has been described in several reports [10–12]. Finally, since our study cohort is supposed to be an extreme sample relative to the general population, it is possible that their initial low values regressed towards their real higher mean platelet levels [13]. However, in most cases the thrombocytopenia persisted without other disorders becoming apparent, suggesting that they may simply represent a part of the left tail of the platelet count distribution observed in healthy individuals [1–3].

Although debatable, our criteria for designating a case as ITP (platelet counts <100 × 10^9^/l) have been already used to determine the incidence of ITP in adults [14], and several authors have applied the definition of “complete response” to ITP patients who achieved platelet counts >100 × 10^9^/l spontaneously or after treatment [15–18]. In fact, the decision to define as ITP a platelet count between 100 × 10^9^/l and 150 × 10^9^/l in an otherwise healthy individual is not supported by recent epidemiological studies. The reference values for platelet counts vary somewhat among laboratories, due to methodology, ethnicity, geographic location, age, etc. Platelet counts below 150 × 10^9^/l have been found in significant proportions of healthy individuals in different geographical areas of the same country [12,19]. Age-related physiological changes are also known to occur, and in a United States National Health and Nutrition Examination Survey (NHANES), the reference values for the elderly were found to be lower than those in younger people [20]. On the other hand, in this study we have shown that individuals with a borderline thrombocytopenia represent a heterogeneous population. Some will develop a more significant thrombocytopenia with the characteristics of ITP, others will develop an autoimmune disease, but the majority will retain a platelet count between 100 × 10^9^/l and 150 × 10^9^/l without developing diseases. Therefore, although there is obviously some degree of overlapping between the two entities, the identification of borderline thrombocytopenia as being different from ITP appears on the whole sustainable from a clinical point of view.

The most frequent event during the initial observation period as well as during subsequent follow-up was the development of an autoimmune disorder. The 10-y probability of developing ITP was 6.9%, whereas that of developing other autoimmune disorders was 12.0%. Since there was no follow-up of the control group, we don't know whether the study cohort has an increased risk of developing an autoimmune disorder. Comparative figures in the general population are not available, although autoimmune diseases have been reported to affect approximately 3% of the US population [5]. Not unexpectedly, in our study most of the cases of autoimmune disease occurred in women. This concords with the prevalence statistics of autoimmune diseases in the general population [21].

The similar prevalence of markers of autoimmunity in the study population and in the control group simply indicates that our cohort was not a selected high-risk subset with regard to these parameters, but does not allow any assumption to be made about the prognostic value of the platelet count itself. As a matter of fact, the results of our study indicate that many cases of autoimmune disease develop in individuals with a low platelet count who do not possess ANA, or other conventional markers of autoimmunity. This is in contrast with the conclusions of a recent retrospective investigation, which implied that autoantibodies are typically present many years before the diagnosis of systemic lupus erythematosus is made [6]. However, in our series there were just two cases of systemic lupus erythematosus (both of whom had detectable ANA on study entry), and in many of the other autoimmune diseases such as psoriatic arthritis or inflammatory bowel disease, there were, in general, no detectable autoantibodies.

In all but one case, the development of an autoimmune disorder was diagnosed because the individuals became symptomatic. Although there were no delays in diagnosis and treatment, the real impact on prognosis of intensive follow-up remains to be demonstrated.

It is noteworthy that many cases considered eligible for the study had chronic thyroiditis, as defined by high titers of TPO-Ab. The relationship between autoimmune thrombocytopenia and chronic thyroiditis is a controversial issue [22,23]. However, the prevalence of chronic thyroiditis in our series of individuals with borderline thrombocytopenia (7%) does not seem higher than in the general population (6.2%), and those with a chronic thyroiditis did not show a higher risk of developing ITP or other autoimmune disorders than the other individuals.

Only four cases of myelodysplastic syndrome were diagnosed, two of which were during the initial 6-mo observation. Therefore the finding of an isolated thrombocytopenia in the elderly in our series was not associated with myelodysplastic syndrome in the majority. This is not surprising, because the reference values for the elderly have been found to be lower than those in younger people [20].

Coronary heart disease and the other diseases that occurred during follow-up, do not appear to be linked to the thrombocytopenia and all patients who developed coronary heart disease were of advanced age with no evidence of vasculitis or other systemic autoimmune disorder. Although the association between immune thrombocytopenia and cancer has been reported in several anecdotal reports and a few small series [24–26], the existence of a pathogenetic link between these diseases is not supported by the rarity of this association.

In conclusion, our study suggests that individuals with a platelet count between 100 × 10^9^/l and 150 × 10^9^/l have a 10-y probability of developing autoimmune disorders of 12%. Further investigation is required to establish whether this risk is higher than in the general population and whether an intensive follow-up of this condition has a positive impact on prognosis.

**Patient Summary**

**Background**

Platelets come from large cells in the bone marrow called megakaryocytes (literally, “cells with large nuclei”). They help form blood clots by releasing substances that activate the blood-clotting process and also by sticking together. Thrombocytopenia (literally, a “lack of thrombocytes”—another word for platelets) is the condition when the number of platelets in the blood drops below the normal range, the lower limit of which is around 150 billion in each liter of blood (written as 150 × 10^9^/l). People with thrombocytopenia may have abnormal bleeding. What is not clear is whether people who have platelet counts that are only slightly lower than normal—between 100 × 10^9^/l and 150 × 10^9^/l—will go on to develop thrombocytopenia, or any other disease.

**Why Was This Study Done?**

The researchers wanted to look at people who had been discovered to have platelet counts between 100 × 10^9^/l and 150 × 10^9^/l (borderline thrombocytopenia) and see what happened to their platelet counts over a long period of time, and whether they developed any diseases.

**What Did the Researchers Do and Find?**

They followed 191 people who had borderline thrombocytopenia. In 64% of those people, the platelet count became normal or stayed low with no other illness. Over 10 years, there was a 6.9% chance of thrombocytopenia and a 12% chance of another autoimmune disorder occurring. Most of the cases of autoimmune disease occurred in women.

**What Do These Findings Mean?**

Other studies have suggested that around 3% of the population will develop autoimmune disease. Hence, having a platelet count just below normal may mean that a person is more likely to develop such an illness, However, since the authors did not directly compare this group of patients with a group of people with normal platelet counts, it is not possible to be sure. So it is not yet clear how worthwhile it is to closely follow people with platelet counts just below normal, and more information is needed on other people with borderline thrombocytopenia.

**Where Can I Get More Information Online?**

MedlinePlus has links to pages of information on thrombocytopenia:

http://www.nlm.nih.gov/medlineplus/ency/article/000586.htm

National Institute of Diabetes and Digestive and Kidney Diseases (NIDDK), which is part of the US National Institutes of Health, has a page of information on thrombocytopenia:

http://www.niddk.nih.gov/health/hematol/pubs/itp/itp.htm

National Guideline Clearinghouse (NGC), a public resource for evidence-based clinical practice guidelines, has a page of information on thrombocytopenia:

http://www.guideline.gov/summary/summary.aspx?ss=15&doc_id=7266&nbr=4328

Dr. Robert McMillan, an expert in platelet disorders, has developed a Web site dedicated to adult chronic immune thrombocytopenic purpura:

http://www.scripps.edu/itp

The ITP Support Association, a UK registered charity that aims to promote and improve the general welfare of patients, and the families of patients, has some pages of information on ITP:

http://www.itpsupport.org.uk

## Abbreviations

ACA: anticardiolipin antibody
ANA: antinuclear antibody
CI: confidence interval
ITP: idiopathic thrombocytopenic purpura
TPO-Ab: antithyroperoxidase antibody
